# 

**Published:** 1876-08

**Authors:** 


					﻿Vol. 23.
AUGUST, J 876.
No. 8.
TWENTY-THIRD YEAR.
— • • -—
HALL’S
Joumal of Health.
PUBLISHED AT 137 EIGHTH STREET, NEW YORK,
Four Doors Fast of 756 Broadway.
Copyright 1870, by E. 11. Gibbs.
AGENTS FOR THE TRADE I
AMERICAN NEWS COMPANY, 121 NASSAU STREET.
NEW YORK NEWS COMPANY, 18 BEEKMAN STREET.
Terms, $2 a Year.
SINGLE NUMBER, 20 CENTS.
John Kent. Printer, 11 Frankfort St., New York.
				

